# Supplementary material for: Effect of Short-Term Restraint Stress on the Expression of Genes Associated with the Response to Oxidative Stress in the Hypothalamus of Hypertensive ISIAH and Normotensive WAG Rats
Source: Antioxidants (Basel). 2024 Oct 26;13(11):1302. doi: 10.3390/antiox13111302 (PMC11590967; doi:10.3390/antiox13111302)
Supplement: Supplementary file 1 [file antioxidants-13-01302-s001.zip › Supplementary Figures S1_S2.pdf]

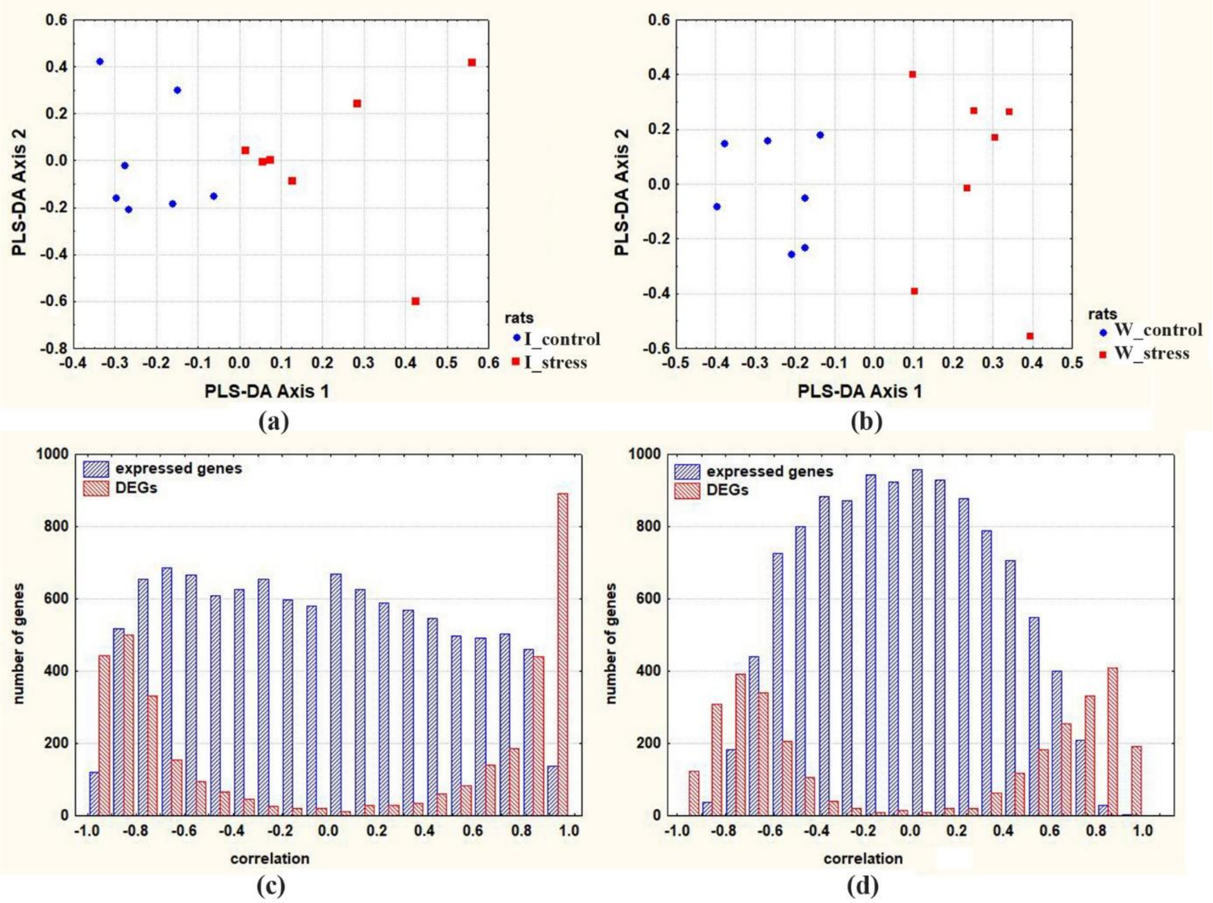

Figure S1. Identification of DEGs contributing most to intergroup differences. (**a**) Distribution of ISIAH rats in the coordinates of the first two PLS-DA axes. (**b**) Distribution of WAG rats in the coordinates of the first two PLS-DA axes. (**c**) Correlation of gene expression with the coordinates of ISIAH rats along the first axis of PLS-DA. (**d**) Correlation of gene expression with the coordinates of WAG rats along the first axis of PLS-DA.

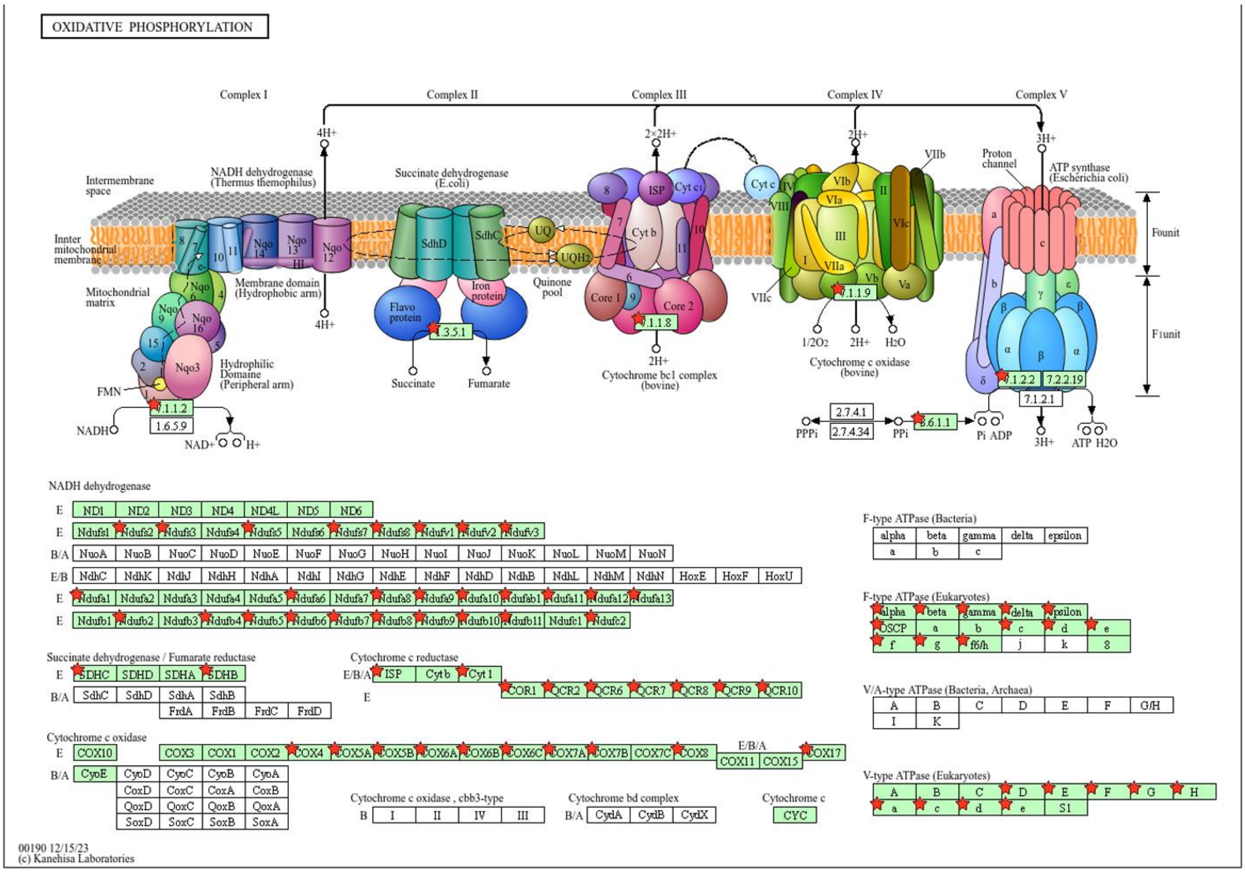

Figure S2. The links of DEGs with the Oxidative phosphorylation pathway (according to KEGG database {Kanehisa, 2023 #1502}). The red stars indicate genes that changed their transcription levels under the influence of a single short-term (2 h) restraint stress in the hypothalamus of hypertensive ISIAH rats.
